# Minimizing Urban Carbon Emissions and Heat Island Intensity: A theoretical study

**DOI:** 10.1371/journal.pone.0330079

**Published:** 2025-09-03

**Authors:** Fabian Reitemeyer, Fabiano L. Ribeiro, Jan Schwarz, Benjamin Gugel, Alexander Schacht, Diego Rybski

**Affiliations:** 1 District Office Friedrichshain-Kreuzberg, Organizational Unit Climate and International, Berlin, Germany; 2 University of Potsdam, Institute of Environmental Science and Geography, Potsdam, Germany; 3 Department of Physics (DFI), Federal University of Lavras (UFLA), Lavras, Minas Gerais, Brazil; 4 Klima-Bündnis der europäischen Städte mit indigenen Völkern der Regenwälder (Alianza del Clima e.V.; Klima-Bündnis e.V.), Frankfurt am Main, Germany; 5 Institut für Energie- und Umweltforschung Heidelberg gGmbH (ifeu), Heidelberg, Germany; 6 Leibniz Institute of Ecological Urban and Regional Development (IOER), Dresden, Germany; 7 Complexity Science Hub, Vienna, Austria; Northeastern University (Shenyang China), CHINA

## Abstract

Cities exhibit both beneficial and detrimental characteristics, many of which stem from agglomeration effects and are, to a first approximation, influenced by population size. However, urban density also plays a critical role. For example, cities with similar population sizes but higher densities tend to emit less carbon, while simultaneously exhibiting a more pronounced urban heat island (UHI) effect. This trade-off highlights the need for a balanced approach that simultaneously minimizes both carbon emissions and the urban heat island (UHI) effect. To address this challenge, we examine how both carbon emissions and UHI intensity are influenced by the population size and spatial extent of the cities. As objective function we define the some of both quantities where city population and area are variables. Considering the scaling relation between area and population as constraint, we derive a theoretical expression leading to an optimal city size. To validate our approach, we analyze carbon emissions data from cities in Germany and consider UHI parameters from the literature. We find that, in the specific case of German cities, achieving an optimal city size that simultaneously minimizes both carbon emissions and UHI intensity is not physically feasible. From a methodological perspective, only the UHI intensity parameters, together with the exponent of the relationship between population and area, determine whether an optimum exists or not. We argue that instead, the scaling relation between population and area itself should be understood as an optimum.

## 1 Introduction

Increasing housing demand in cities can be satisfied by *urban expansion* or by *urban consolidation* (aka densification) [1]. Urban expansion is characterized by new urban development at the edges or fringes of the considered cities [2]. Urban consolidation is characterized by development within existing urban boundaries, typically by increasing population density through infill or vertical development [3]. While urban expansion affects the non-urban surroundings and spares the urban centers, urban consolidation does the opposite, sparing the non-urban countryside and affecting the urban areas. Since urban expansion is associated with problems related to sprawl [4, e.g.] and phenomena such as the wildland-urban interface [5, e.g.], in many cases, urban consolidation represents the preferable choice, besides coming with advantages and disadvantages [6]. Benefits of urban consolidation include decreased travel distances and reduced pressure on land and land-take [7, e.g.]. However, urban consolidation problems arise when public spaces are repurposed, and in dense urban environments, it becomes challenging to preserve green spaces [8].

In view of climate change and efforts towards sustainable urban development, urban planners face the dilemma of urban expansion vs. urban consolidation [9, e.g.]. Climate change mitigation calls for a reduction in urban carbon emissions. High densities in consolidated cities foster a shift in modal mobility towards public and active transport, and emissions are lower due to shorter distances. However, high population densities in cities increase the urban heat burden [10,11, e.g.] and subsequent morbidity and mortality [12] along with adverse other health effects [13]. Urban densification can pose a threat to urban green space [8], and cities exhibiting lower population density are greener [14]. Thus, a trade-off is necessary, balancing the benefits and drawbacks of both strategies.

We approach the problem of assessing urban expansion and consolidation theoretically. To be more precise, we do not consider any dynamics but rather seek a static, optimal compromise. Specifically, we consider two quantities that we want to optimize: urban carbon emissions and urban heat island (UHI) intensity. Moreover, we use *city population* and *city area* as variables that can be tuned, for example, by urban planning policies. Combining both implicitly involves the density [15]. As the last ingredient, we include the well-established scaling relation between population and area, which we consider a constraint. In summary, we derive an expression for the optimal city size (i.e., optimal area and population pair), which simultaneously minimizes carbon emissions and heat island intensity.

Most studies on the relationship between urban carbon emissions and heat island effects investigate the association between UHI intensity, cooling energy, and related carbon emissions. E.g. for the USA, it was found that UHI significantly increases the energy bill for states in warm climates, while for some regions located in cold climates, it is the opposite [16]. Similarly, the effectiveness of urban green space in reducing UHI and carbon emissions was analyzed [17]. A somewhat different empirical approach was pursued in [13], where low UHI were found in green low density cities and contrasting low per capita CO2 emissions in compact high density cities. Our work theoretically explores this incompatibility of low UHI and low carbon emissions. Another approach consists of spatial optimization within cities [18,19], where spatial configurations of land-use or local climate zones are optimized with respect to multiple objectives, including climate impacts.

As an empirical case, we use carbon emissions data from the *Klimaschutz-Planer* (KSP), a German platform used by municipalities to do their emissions inventories. KSP is an internet-based software for balancing greenhouse gases. It actively implements the municipal balancing system (BISKO) and is the most widely used balancing tool at the municipal level. The final energy consumption in BISKO is assessed according to the territorial principle (Scopes 1 and 2, see [20,21, e.g.]). In other words, the final energy consumption that occurs within the boundaries of the municipalities is included. The advantage is that the data entered by the municipalities is processed using the same methodology (KSP), and the resulting estimates are consistent and comparable. We use a bivariate scaling approach (emissions as a function of population and area) to describe the total emissions of German cities. For the UHI part, we use parameters from the literature that are specific to the Berlin background climate [10].

From the proposed model, we find that combining the exponents and parameters of urban carbon emissions and the UHI intensity leads to optimal values of the city area and the population that are physically not possible (i.e., not real-valued). We conclude that if a decision-maker were to retrofit their city to minimize carbon emissions and UHI intensity, they would not be able to do so.

The search for an optimal city size has a long history in the scientific literature, particularly in urban economics. The idea behind optimal city size is that beyond such a size, agglomeration advantages decrease [22]. E.g., Arnott [23] employs a utility-maximizing framework using residential location theory, where space and transportation costs are considered. More recently, Camagni et al. [24] combine traditional urban economics with other determinants, such as environmental quality. Instead of a general optimal size, the authors arrive at a city-specific “equilibrium” size. Capello & Camagni [22] argue that there is a contradiction between the theoretical optimal city size and the real-world development of urban systems. Consequently, the authors explore other determinants than the size of the city and conclude that *efficient* size should replace the optimal size. Our work makes a humble contribution to the field, employing a form of urban scaling and questioning the existence of an optimal urban size. To the best of our knowledge, previous approaches never optimized carbon emissions and UHI intensity and also did not take scaling relations into account.

## 2 Urban Carbon Emissions

Understanding how carbon emissions scale with urban characteristics is essential for developing effective mitigation strategies. In particular, the interplay between a city’s population and its spatial extent plays a central role in determining its overall emissions profile. In this section, we combine city population size and area to model carbon emissions, following an approach similar to that explored in [15], which conceptualizes emissions through production functions. The simplest form is an expression resembling the Cobb-Douglas model [15]

$$C=c_{1}P^{\beta_{P}}A^{\beta_{A}},$$ (1)

where $\beta_{P}$, $\beta_{A}$, and *c*_1_ are obtained from fitting to the data. The case $\beta_{P}=\alpha +1$ and $\beta_{A}=-\alpha$ corresponds to $\frac{C}{P}\sim (\frac{P}{A}{)}^{\alpha }$ and $\beta_{P}+\beta_{A}=(\alpha +1)+(-\alpha )=1$ implies constant returns to scale [15]. In a more general context, the condition $\beta_{P}+\beta_{A}>1$ implies increasing returns to scale—meaning that carbon emissions grow faster than city size (area and population). Conversely, $\beta_{P}+\beta_{A}<1$ indicates economies of scale, in the sense that per capita carbon emissions decrease as city size (area and population) increases. Accordingly, Eq (1) represents a generalization, implicitly containing the density.

We analyze data from more than 300 municipalities in Germany where carbon emissions have been assessed in a standardized way (see Materials and Methods). The data comes in administrative boundaries so that we use population and area values for the same spatial delineations.

**Fig 1:**
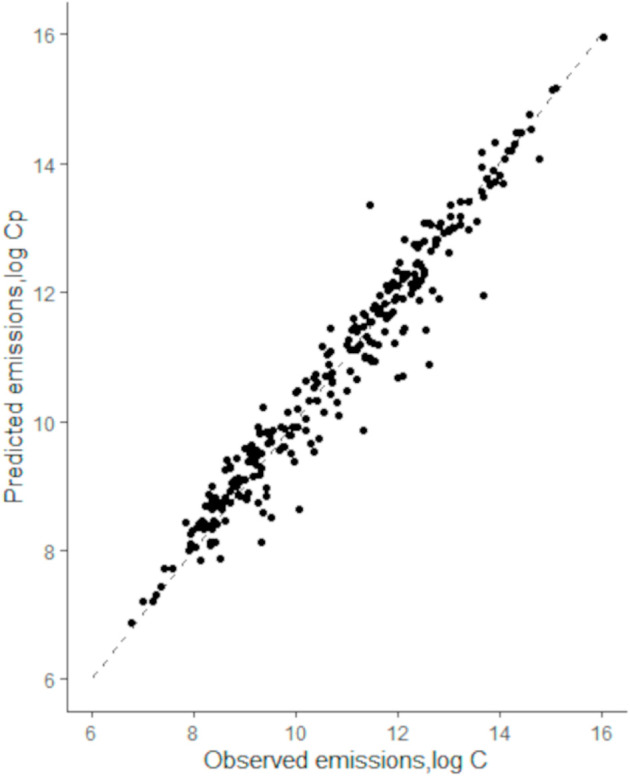
Urban carbon emissions in German cities. Predicted, from Eq (1), vs. observed values. The dotted line corresponds to a diagonal and represents equality.

Our results for total carbon emissions are shown in Fig 1, where we plot the predicted values from Eq (1) vs. observed values. We see a fair agreement between prediction and observation. The estimated parameters are listed on Table 1. As $\beta_{P}+\beta_{A}=(1.056-0.003)>1$, we conclude increasing returns to scale, slightly deviating from the urban scaling result with β≃1 (not shown). We note, however, that Eq (1) is also a generalization of $C\sim P^{\beta }$, but because $\beta_{A}$ is only slightly different from 0, also the difference to conventional urban scaling is minor.

**Table 1:** Overview of exponents and parameters. For the symbols in the first column, the estimates are listed in the second column, with standard error if available (3rd). The fourth column lists the equations in which the parameters are introduced. The last column provides the literature reference for those estimates which do not stem from our own analysis.

Parameter	Estimate	Std. Error	Equation	Source
$\beta_{P}$	1.056	0.021	(1)	
$\beta_{A}$	−0.003	0.001	(1)	
*δ*	0.939	0.020	(4)	
*b*_*P*_	0.65		(2)	[10]
*b*_*A*_	−0.43		(2)	[10]
*c*_1_	1.831	0.176	(1)	
*c*_2_	3.9		(2)	[10]
*c*_3_	0.001	0.174	(4)	

## 3 Urban Heat Island Intensity

The UHI intensity ΔT is given by the temperature difference of a city $T_{\text{C}}$ compared to its non-urban surroundings $T_{\text{B}}$, i.e. $\Delta T=T_{\text{C}}\;-\;T_{\text{B}}$. Interestingly, a similar form to the Cobb-Douglas model has been reported for the UHI intensity [10, Eq (1)] (Originally, [10, Eq (1)] is $\Delta T=b_{P}\ln S+b_{A}\ln A+c_{2}^{\prime }$ where *S* is the total building volume and conversion to population *P* leads to *c*_2_ that only differs by a factor from $c_{2}^{\prime }$.):

$$\Delta T=b_{P}\ln P+b_{A}\ln A+c_{2}\;\text{ with }b_{A}<0,$$ (2)

where *b*_*P*_, *b*_*A*_, and *c*_2_ are parameters. Please note that the only parameter expected to be negative is *b*_*A*_, i.e., the UHI intensity decreases with increasing city area (when the population is kept constant). With increasing area, while keeping the population constant, the population density decreases, there is more space between the buildings, and consequently the UHI intensity decreases. Accordingly, the parameter *b*_*A*_ must be negative as reported in [10].

We use the values reported in [10] as listed in Table 1. To our best knowledge [10] is the only study providing the parameters *b*_*P*_, *b*_*A*_. They are specific to the background climate of Berlin and accordingly, the interpretations and applicability of these parameters are limited to Berlin.

## 4 Trade-offs and optimization

We want to minimize both the carbon emissions and the UHI intensity. This can be done by optimizing the sum of both. Adding up both, we are comparing apples and oranges so that we introduce weights *w*_1,2_>0, with appropriated physical units, relating the two quantities

$$B=w_{1}C+w_{2}\Delta T,$$ (3)

where *C* and ΔT are both functions of *P* and *A*. If we want to minimize the objective function *B*, we cannot ignore the fact that more populous cities also occupy more space. This is reflected in the *fundamental allometry* [25–27]

$$A=c_{3}P^{\delta }\;$$ (4)

where *δ* is another exponent and δ<1 is reported in most cases [28,29]. The relation between the variables *A* and *P* represents a constraint for the minimization of *B*.

Employing the *Lagrange Multiplier* to find the optimal area (and population) that promotes this minimization (see Materials and Methods), we obtain

$$A_{\text{opt}}={\left[-\frac{w_{2}}{w_{1}}\frac{(\frac{b_{P}}{\delta }+b_{A})c_{3}^{\frac{\beta_{P}}{\delta }}}{\left(\frac{\beta_{P}}{\delta }+\beta_{A}\right)}\right]}^{\frac{1}{\beta_{A}+\frac{\beta_{P}}{\delta }}}.$$ (5)

Considering only the values of *A* and *P* that obey the fundamental allometry, $A_{\text{opt}}$ and $P_{\text{opt}}=(A_{\text{opt}}/c_{3}{)}^{1/\delta }$ are the area and the population that minimize *B*, given a choice of *w*_1,2_>0.

Eq (5) looks more complicated than it is. An optimum exists only if the big parenthesis [·] is positive – otherwise, the power function has no real-valued result. Thus, another negative sign needs to compensate the negative sign inside the parenthesis. Since it only includes addition, the negative sign must come from the parameters. The only negative parameter is *b*_*A*_. Thus, there is an optimum if the round brackets in the numerator is negative, $(\frac{b_{P}}{\delta }\;+\;b_{A})<0$, i.e. $\frac{b_{P}}{\delta }<-b_{A}$. In our case, taking the values from Table 1, we obtain $\frac{b_{P}}{\delta }≃0.69$ and $b_{A}≃-0.43$, i.e. 0.69 > –(–0.43), so the big parenthesis [·] is negative. Consequently, there is *no optimum* in *B* and no optimal city size (given our set of parameters, which is restricted to German cities). This finding is independent of the choice of the weighing parameters *w*_1_ and *w*_2_.

Unfortunately, we do not have error estimates for the parameters *b*_*P*_ and *b*_*A*_. To address this limitation, we assume a conservative error margin of 20 % for both parameters. This implies that the minimum expected value for *b*_*P*_ is $b_{P}(1\;-\;0.2)=0.520$, and the minimum expected value for *b*_*A*_ is $b_{A}(1\;+\;0.2)=-0.516$. Additionally, considering that δ=(0.94±0.02), we take its maximum value, δ=0.96. Substituting these extreme values into the expression $\left(\frac{b_{P}}{\delta }+b_{A}\right)$ yields its minimum possible value, which still remains positive. Consequently, the term inside the large brackets in Eq (5) remains negative, even under these extreme assumptions. This indicates that a non-trivial optimum does not exist, even when accounting for a 20 % uncertainty in the parameters *b*_*P*_ and *b*_*A*_.

## 5 Discussion

In summary, we investigate the optimal city size that minimizes urban carbon emissions (Germany) and UHI intensity (Berlin’s background climate). To this end, we use functional forms dependent on city population and area, with the fundamental allometry between them serving as a constraint. (i) We derive a theoretical expression for the optimal size using the Lagrange Multiplier method. (ii) Comparing this expression with the estimated exponents and parameters reveals that the conditions imply the absence of a non-trivial optimum city size.

It is not possible to achieve the theoretical optimal city size, as the estimated parameters indicate that carbon emissions increase with area more rapidly than UHI intensity decreases with it. Even if cities cannot be fully optimized in terms of carbon emissions and UHI intensity, urban planners can still pursue strategies to reduce these outcomes. This is because Eqs (1) and (2) are based on regressions, and individual cities can lie above or below the predicted trends—ideally below. Moreover, reductions in emissions and UHI intensity could contribute to lower values of the coefficients *c*_1_ and *c*_2_, yielding broader environmental benefits. Even if an optimal city size were theoretically attainable, it would not be feasible for planners to substantially modify population or area to reach it.

In this paper, the two quantities, urban carbon emissions and UHI intensity, serve as examples of counteracting forces in cities. Of course, the population and area of cities are the result of many factors, many of which are beyond the control of decision-makers. However, conflicting goals are common in urban planning and our results show that an optimal solution is not always found, even for specific factors. In addition, it is unsurprising that we do not find an optimal city size. If there was some optimum, and if we assume that decision makers try to improve cities, then the cities should at least move towards such an optimal size. But this is not what we observe in reality. Most cities around the world follow a power-law distribution of population size [30,31, e.g.] – essentially, a distribution without a typical size, which implies the absence of a typical or optimal city size toward which urban development could converge. In this sense, rather, the fundamental allometry Eq (4) should be understood as an optimal relation between area and population [27].

Interestingly, whether there is an optimal city size or not depends only on the parameters of the UHI intensity, $b_{A},b_{P}$, and *δ* (the exponent of the fundamental allometry) – but not on the exponents of the carbon emissions ($\beta_{A}$ and $\beta_{P}$). Although the obtained parameters do not lead to an optimum, we can ask under which conditions this would be the case. The inequality $(\frac{b_{P}}{\delta }\;+\;b_{A})\;<\;0$ implies that there is a critical exponent $\delta_{\text{c}}=\frac{b_{P}}{-b_{A}}≃\frac{0.65}{-(0.43)}≃1.51$ that is at the onset of a regime where such an optimum $A_{\text{opt}}$ exists. However, this critical value is not only larger than 1 – while δ<1 is reported in most cases – with a value much larger than 1 it is also strongly super-linear. This means that an unrealistic large *δ* would be required for an optimal city size (given the parameters $b_{A},b_{P}$). For other cities or countries, we expect different estimates and possibly different conclusions.

For the UHI part, we need to rely on the values provided by [10], which are limited to the background climate of Berlin. The authors used urban climate modeling and replaced urban morphology with a wide range of generated city structures to arrive at Eq (2). In this way, the authors avoided the problem that in studies across cities, two components always vary simultaneously, namely the urban morphology *and* the background climate. The authors of [10] managed to keep the latter constant, but a complementary analysis (the same morphology in different climates) is pending. Such a complementary analysis will likely affect the estimated parameters and thereby the outcome of our conclusions. Additional studies providing estimates of the UHI parameters *b*_*P*_ and *b*_*A*_ could mitigate the data limitations of the work in hand.

Of course, the definition of a city also plays a role. Depending on the choice of city boundaries, urban scaling exponents can change from sub- to super-linear [32]. It cannot be excluded that the estimates in Eq (1) can be affected similarly. KSP uses administrative city boundaries and in the UHI case [10] an urban cluster definition was used [33].

In terms of carbon emissions, it is obvious that the emissions of a city also depend on the presence or absence of carbon-intensive industry. Here, we do not take the composition of the economy into account [34, e.g.]. We assume that it would solely increase the residuals of the regression Eq (1). Anyways, given that we would obtain a better description of the carbon emissions, it would not affect our conclusions as the parameters of the UHI intensity are decisive for the optimum. In addition, with regard to the urban climate, more detailed data could improve the description [35, e.g.].

Another point that merits discussion concerns the additive formulation adopted in Eq (3), where the objective function *B* is defined as a weighted sum of carbon emissions (*C*) and urban heat island intensity (ΔT). This linear combination is intuitive and facilitates straightforward interpretation. However, alternative formulations are also plausible. For instance, one could adopt a multiplicative or ratio-based form, which would emphasize relative rather than absolute changes in the two quantities. Each of these alternatives reflects different assumptions about the trade-off between emissions and heat intensity, and would yield distinct optimization outcomes. As such, the choice of functional form should be informed by both empirical behavior and policy priorities.

Here, we combine power-laws of area and population with logarithmic functions. This is because the value range of UHI intensity is not as broad as that of carbon emissions. In fact, the precise question of how the intensity of the UHI depends on the size of the city remains unresolved. For surface temperature, it seems to saturate [11], but for air temperature (as in this study), the relation could be logarithmic (as in [10]) or power-law. Even in the case of a power-law, the exponent is small, and the value range of UHI intensities remains small compared to the respective population values. Thus, if other quantities were to be compared in future work, which has the power-law form as Eq (1), a different derivation would be necessary. Our work is also limited to only two factors, and arbitrary many could be included in the objective function.

## Materials and methods

### Data

The data used is based on the “Bilanzierungs-Systematik Kommunal” (BISKO), which serves to calculate municipal greenhouse gas emissions using a uniform methodology. The basis of this methodology is the territorial approach (Scope 1). All emissions within the considered geographical boundaries are taken into account.

Given the local scope of application and the municipalities’ low influence on the area’s businesses, this approach was extended to include the end-energy-based territorial balance. This means indirect emissions generated outside the territory are also considered, i.e., stemming from electricity, heat, and steam that occur during the generation at the supplier’s premises.

The *Klimaschutz-Planer* (KSP, https://www.klimaschutz-planer.de/) is an internet-based software for balancing greenhouse gases and is operated by the Klima-Bündnis. It actively implements BISKO and is the most widely used accounting tool at the municipal level in Germany. Using the Klimaschutz-Planer incurs costs that depend on the number of inhabitants in the municipality (in this case, the district). It provides a comprehensive greenhouse gas accounting.

The dataset obtained from KSP contains municipality-specific information and calculated carbon levels from 2014-2018. In this paper, we selected data from 2017, the year with the largest number of municipalities (357). Municipality-specific information includes population size, local electricity grid emission factor [t/MWh], official municipality key, and data score. Carbon emissions [tCO2eq] are based on individual sectors’ final energy consumption [MWh]. The sectors specified are heat, electricity, industry, municipal facilities (buildings), private households, trade, commerce, services, other, and transport.

Population data was available for all 357 municipalities analyzed; information on population is usually preset in the KSP and is based on statistical data. They refer to the administrative boundaries.

Area data for cities was extracted from CORINE land cover 5 (CLC5) data for the year 2018 to calculate their density. The dataset CLC5 represents a landscape description in vector format according to the nomenclature of CORINE Land Cover (CLC), which reflects the land cover based on the aspects of land use. The CLC5 is derived from the Land Cover Model Germany 2018 (LBM-DE2018) [36], which differentiates between land cover (LB) and land use (LN), as well as information on the proportion of sealed land (SIE) and vegetation (VEG) for a minimum object size of 1 ha. In order to link the area with the municipalities in our database, a Germany-wide map with the administrative boundaries and the corresponding municipality keys was intersected with the CLC5 information. Subsequently, the areas could be linked to the data available to us on the basis of the municipality keys. Population density is then simply given by the population divided by the area [km^2^].

### Optimization employing the Lagrange Multiplier

To deal with the trade-off between urban carbon emissions and UHI intensity, let us define a function *B*(*P*,*A*) for a city with a population *P* and Area *A*. This quantity depends linearly on the city’s carbon emission, say *C*(*P*,*A*), and its UHI intensity ΔT(P,A), in the form

$$B(P,A)=w_{1}C(P,A)+w_{2}\Delta T(P,A).$$ (6)

According to empirical evidence

$$C(P,A)=c_{1}P_{P}^{\beta }A^{\beta_{A}},$$ (7)

and

$$\Delta T(P,A)=b_{P}\ln P+b_{A}\ln A+c_{2}\;.$$ (8)

The parameters $\beta_{P},\beta_{A},b_{P},b_{A},c_{2}$ are obtained from data, but *w*_1_ and *w*_2_ are parameters that depend on public policy and reflect necessities and priorities. In addition, given that ΔT decreases for a larger area, it is necessary that *b*_*A*_<0.

We want to investigate if there is some optimal value for *P* and *A* that minimizes *B*(*P*,*A*). In other words, we want to know what combination of size and area will simultaneously minimize the carbon emission and UHI intensity.

In addition, the area of a city is strongly dependent on the population size, following the so-called fundamental allometry, which states a power-law relation between these two quantities as

$$A=c_{3}P^{\delta }$$ (9)

where δ<1 and *c*_3_ are parameters that can be obtained from data. In this way, the problem we have is finding *A* and *P* that minimize *B*, say *A*_*c*_ and *P*_*c*_, given the constraint Eq (9).

The methodology appropriated to deal with this situation is the *Lagrange Multiplier* technique. Following the methodology, we need to solve the equation

$$\vec{\nabla }B(A_{c},P_{c})=\lambda \vec{\nabla }h(A_{c},P_{c})$$ (10)

where

$$h(A,P)\equiv A-c_{3}P^{\delta }=0$$ (11)

is the constraint, and *λ* is the Lagrange Multiplier. The nabla operator (or the gradient) is given by

$$\vec{\nabla }(\cdots )=\frac{\partial (\cdots )}{\partial A}\hat{ı}+\frac{\partial (\cdots )}{\partial P}\hat{J}$$ (12)

To solve Eq (10) means to solve the following two differential equations

$$\frac{\partial B}{\partial A}{)}_{\left(A_{c},P_{c}\right)}=\lambda \frac{\partial h}{\partial A}{)}_{\left(A_{c},P_{c}\right)}$$ (13)

and

$$\frac{\partial B}{\partial P}{)}_{\left(A_{c},P_{c}\right)}=\lambda \frac{\partial h}{\partial P}{)}_{\left(A_{c},P_{c}\right)}$$ (14)

Inserting one equation into the other by the *λ* multiplier, one has

$$\frac{\frac{\partial B}{\partial A}{)}_{\left(A_{c},P_{c}\right)}}{\frac{\partial h}{\partial A}{)}_{\left(A_{c},P_{c}\right)}}=\frac{\frac{\partial B}{\partial P}{)}_{\left(A_{c},P_{c}\right)}}{\frac{\partial h}{\partial P}{)}_{\left(A_{c},P_{c}\right)}}.$$ (15)

The point $\left(A_{c},P_{c}\right)$ which we are looking for (the one that maximizes B given the constraint given by the fundamental allometry) must obey the relation Eq (15).

These derivatives can be done easily using Eqs (6) and (11), which yields

$$\frac{\partial h}{\partial A}{)}_{\left(A_{c},P_{c}\right)}=1$$ (16)

$$\frac{\partial h}{\partial P}{)}_{\left(A_{c},P_{c}\right)}=-c_{3}\delta P_{c}^{\delta -1}$$ (17)

$$\frac{\partial B}{\partial A}{)}_{\left(A_{c},P_{c}\right)}=\beta_{A}w_{1}c_{1}P_{c}^{\beta_{P}}A_{c}^{\beta_{A}-1}+\frac{w_{2}b_{A}}{A_{c}}$$ (18)

$$\frac{\partial B}{\partial P}{)}_{\left(A_{c},P_{c}\right)}=\beta_{P}w_{1}c_{1}P_{c}^{\beta_{P}-1}A_{c}^{\beta_{A}}+\frac{w_{2}b_{P}}{P_{c}}.$$ (19)

Inserting these four derivatives in the Eq (15), an area *A*_*c*_, and consequently a population $P_{c}=(\frac{A_{c}}{c_{3}}{)}^{\frac{1}{\delta }}$ (given by the constraint), that minimize *B*(*P*,*A*), and its value is

$$A_{c}={\left[-\frac{w_{2}}{w_{1}}\frac{(\frac{b_{P}}{\delta }+b_{A})c_{3}^{\frac{\beta_{P}}{\delta }}}{\left(\frac{\beta_{P}}{\delta }+\beta_{A}\right)}\right]}^{\frac{1}{\beta_{A}+\frac{\beta_{P}}{\delta }}}.$$ (20)

Considering only the values of *A* and *P* that obey the fundamental allometry, *A*_*c*_ and *P*_*c*_ are the area and population that minimize *B*(*P*,*A*).
